# Understanding interaction in problematic dementia and social care encounters: Protocol for a micro-level study combining video-ethnography and Conversation Analysis (CA)

**DOI:** 10.1371/journal.pone.0305069

**Published:** 2024-06-14

**Authors:** John Chatwin, Katherine Ludwin, Danielle Jones, Alison Bravington

**Affiliations:** 1 Midlands Partnership NHS Foundation Trust, Hebden Bridge, United Kingdom; 2 Centre for Applied Dementia Studies, University of Bradford, Bradford, United Kingdom; 3 Wolfson Palliative Care Research Centre, Hull York Medical School, University of Hull, Kingston upon Hull, United Kingdom; PLoS ONE, UNITED STATES

## Abstract

**Introduction:**

It is well established that the actions and behaviour of dementia care workers are fundamental to the wellbeing of the people they care for. Not only do they deal with basic healthcare needs, but they also perform a vital psycho-social function by providing–through their regular presence–an underlying continuity for residents. This has been shown to improve well-being, particularly for those in the advanced stages of dementia. It has also been suggested that there are additional psycho-social benefits of such contact which can directly influence the need for anti-psychotic medication. However, unlike most other healthcare and medical settings, the specialised and often difficult interactions that dementia care workers handle every day have not yet been the subject of detailed micro-level analysis. This is particularly significant because much of the impact that care-workers have relates to the way in which they interact with the people they care for. Not having a clear understanding of how their interactions ‘work’ at the micro-level–particularly ones that are specific to dementia care settings, and that care workers report to be difficult or challenging–means that any training interventions that are developed may not resonate with their real-world experience, and ultimately run the risk of failing. This video-based observational study aims to provide a detailed micro-exploration of problematic and challenging interactions involving care-workers and people living with dementia.

**Setting and methods:**

The study is based in the UK and will involve up to 20 dementia care staff and 60 people living with dementia. Fieldwork will be conducted in 5 dementia care home and community-based dementia day care settings using naturalistic observational methods (primarily video-ethnography). Data will be analysed using Conversation Analysis (CA).

## Introduction

Recent years have seen major UK policy initiatives aimed at improving residential and dementia-related care, at both a medical and psycho-social level. Significantly, emphasis has always been placed on research which addresses day-to-day issues for people in long term care. Ground level care staff are centrally implicated in this and understanding their role and activities needs to be high on the research agenda.

Despite the importance of their role, care workers have always been a marginalised sector of the healthcare workforce; they face one of the most challenging roles but are frequently marginalised, under-trained, and undervalued [1]. Much research in this area has concentrated on negative aspects of the role, such as institutional abuse, staff-burnout, and job stress [2]. Similarly, although care work involves many nursing related tasks, it is often perceived as a demeaning ‘last resort’ by nursing professionals [3]. This culture contributes to the high staff turnover that is a feature of the sector and this can, in turn, undermine continuity and stability for residents–an important factor in the maintenance of their wellbeing [4–6].

The importance of the care worker role is increasingly acknowledged, and there is a growing body of research specifically aimed at the care home environment [7]. However, much of this has been broadly ethnographic and focused on the implementation and effectiveness of discrete interventions. Interactional ‘high points’ such as mealtimes, for example, have been the subject of several studies [8]. Research has also examined the effect of care staff shift patterns on resident wellbeing [9], and coding of staff-resident interaction has been utilised as a direct measure of quality of care [10].

Although a range of care worker/resident issues have been explored, there are few interactional studies providing a more detailed micro-level analysis of dementia care interaction. Notable exceptions being work analysing the way carers formulated talk about aggressive patients [11] and examining how healthcare professionals manage the closure of encounters with people with dementia in hospital settings [12]. Some studies have addressed verbally disruptive behaviours and problematic communication patterns between people with dementia and their family carers [13, 14]. Similarly, others have provided a micro-level analysis of interactional difficulties arising from dementia related memory issues in family encounters and couples’ dementia therapy sessions [15, 16]. However, apart from one study offering a micro-analysis of how care workers helped residents who were experiencing episodes of confabulation [17], micro-level work dealing with the kinds of challenging situations we are concerned with is scarce. This is significant because–particularly in care home settings–difficult or badly resolved situations are likely to be potent accumulators of well or ill being for residents and staff alike.

## Aims and objectives

In the context of this study a ‘difficult’ interaction is regarded as any encounter that arises out of, or appears to cause, a degree of interactional misalignment to interlocutors. This may range from minor dementia-related communication problems and misunderstandings, right through to more challenging physical situations that care staff may need to manage.

*The study aims to*:

i) Provide a systematic micro-level analysis of challenging and difficult interactions that professional care workers and family carers often face while caring for a person with dementia.

ii) Determine the key micro-level features that influence how these interactions develop, how they play out, and how they are resolved.

iii) Work collaboratively with professional care workers to produce exploratory video-based training resources, based on the study findings.

## Research plan

### Setting

The study will take place in 5 dementia care home and community day-care settings, operated by local councils, private companies, and voluntary and charitable organisations. The sites represent a cross-section of current dementia care-home and community support environments where care staff are employed.

### Sample

The makeup of individual research sites will influence the number of participants involved. As an approximate guide, based on previous studies conducted by the research team [18–20], and comparable video-ethnographic / CA based studies [21, 22], we aim to include up to 20 care-staff and 60 residents and other stakeholders in the study.

### Recruitment (care staff participants)

We will work closely with home managers to identify staff participants. Well in advance of fieldwork, we will raise awareness of the project by displaying posters at fieldwork sites and meet with staff to explain the project. We will obtain preliminary consent from staff during this phase, and then utilise these contacts to identify residents who might be willing to participate.

### Recruitment of residents and other stakeholders

The specific dates, times, and location where fieldwork is due to take place in individual sites will be agreed with care staff and managers. Information leaflets tailored for each location will be sent to family carers of all residents outlining when researchers will be attending, and what this will involve. Family members will be encouraged to get in touch with the research team to discuss any issues that may concern them, and time has been factored in to the research plan to accommodate this. Residents who have capacity to give informed consent will be approached by a member of the research team or care-staff during a preliminary site visit. The study will be explained to them and provisional consent requested. They will also be given a clear information leaflet about the study.

### Participants without capacity

For participants who do not have capacity to give informed consent, we will follow the 2005 Mental Capacity Act guidelines and seek advice from a consultee to ascertain what the wishes and feelings of the person might be, and whether or not they are likely to have agreed to take part prior to losing capacity. Consultees will initially be identified via the care-home and given the relevant information sheet. A member of the research team will follow this up three or more days later and answer any questions they may have. They will then be asked to sign a consultee declaration form to indicate that they feel the person they are representing would have wished to take part in the study.

### Ongoing / process consent

**Fig 1:**
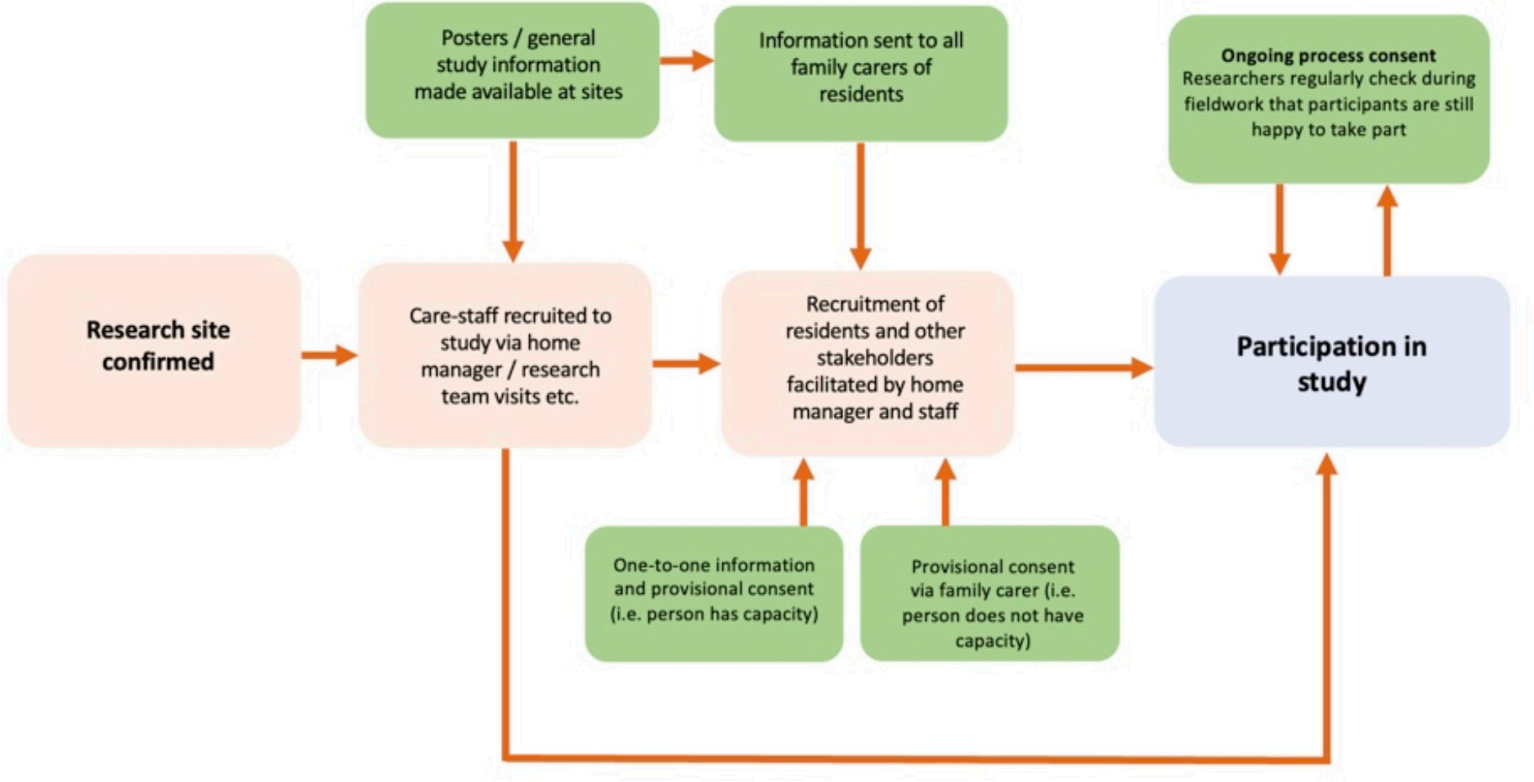
Recruitment and consent process.

Because provisional consent will be taken in advance of actual fieldwork, residents–even those who do not necessarily have severe cognitive problems–may not remember that they have agreed to take part in the study. Therefore, once fieldwork is under-way, a *process consent* system will be used to continually check that people are happy being filmed and taking part in the study (See Fig 1).

### Inclusion and exclusion criteria

The study will include full-time, part-time, and voluntary care workers, visiting family carers, and any other stakeholders who engage with residents at the study sites. Any care home residents themselves living at or attending a site may also take part. The principal inclusion criteria is simply that a person is willing to engage with the study and consent to have their natural interactions videoed. There are no exclusion criteria for staff, care home residents or other stakeholders.

## Methods

The study will use a combination of participatory and non-participatory observation (primarily naturalistic video recording), Conversation Analysis (CA), and informal ad hoc interviews with carers, residents, and other stakeholders. Where possible, these will involve a ‘stimulated recall’ process [23], which has been adapted specifically for use in dementia care environments [24].

### Conversation Analysis (CA)

CA is well established as a highly effective method for investigating interaction in a wide variety of settings. It is largely concerned with the analysis of the verbal communicative practices that people routinely use when they interact with one another. Essentially, CA uses video and audio recordings of naturally occurring interaction, and a highly detailed method of transcription which aims to capture the minutiae of speech and aspects of non-verbal behaviour. It provides an analytical method that can expose the underlying ‘rules’ that govern how activities are composed and organised [21]. As well as being an academic discipline that can be applied in isolation to reveal a level of interactional detail often inaccessible using other methods, CA has a long track record of work in applied fields.

In the area of medical interaction, CA has been used to investigate primary and secondary care consultations [25], health visiting [26], counselling [27], mental health [28] and complementary and alternative medical settings [29–32]. Studies have concentrated on providing a broad analysis of particular clinical environments [33–35], and work has also focused on exploring specific aspects of interaction within these settings–such as the ways in which patients ‘frame’ their presenting complaints, and the way this influences clinician responses [36–38].

Although research utilising socio-linguistic techniques to investigate care-worker interaction in general is by no means absent [39–41], the field has received significantly less attention than other areas of health and social care. While doctor / patient interaction has a well-established research tradition, studies focusing specifically on dementia care-home staff and their residents are scarce. A number of ethnographic studies have incorporated elements of talk-based analysis [42, 43]. However, very little work with CA as a primary methodology has been completed.

### Observation and interviews

CA will be used alongside broader qualitative methods: non-participant and participant observation, and informal interviews with participants that incorporate the principles of ‘stimulated recall’ [44]. This approach will enable practical connections between the micro-level analysis that CA provides and the ‘real world’ of the care-worker to be developed.

## Research plan

This is an 18-month study, broadly comprised of 4 phases:

### Phase 1: Pre-study/preparation

#### Months 1–4

This includes identifying potential research sites; drafting an NHS ethics application; preliminary site-visits and recruitment of participants; fieldwork planning, co-ordination, and technical orientation.

### Phase 2: Fieldwork / data-collection

#### Months 4–11

Fieldwork will be conducted over a period of approximately 7 months. Flexibility on exact arrangements will be required in order to accommodate the access requirements of individual sites, and the working patterns of care-staff etc. It is anticipated that up to 8 fieldwork visits will be made to each site over the course of the study.

#### Data collection

The primary aim of the fieldwork is to video as many *naturally occurring* ‘difficult’ interactions between care-workers and residents as possible. To capture these, we will utilise two complementary video-ethnographic approaches. Depending on the specific characteristics of a site, these may be used individually or in combination.

#### Approach 1 –remotely monitored static cameras

This approach is particularly suited to capturing the kind of interactions we are interested in as it is unobtrusive and creates minimal disruption to the care environment. We will work with care-home staff to identify communal spaces, such as lounge or activity areas, that can be informally demarcated for filming. We will then set up small ‘GoPro’ cameras, as well as mounted camera phones and iPads. To minimise disruption, if required, cameras will be monitored by Wi-Fi from elsewhere in the building and controlled remotely.

#### Approach 2—mobile camera–semi-participatory

This is a well-established video-ethnographic approach that has been used in a variety of dementia-related studies [45–47]. It involves a researcher working one-to-one with individual care-staff, essentially accompanying them with a small video-camera as they go about their activities. This method works well in care-home and dementia environments because if used sensitively over an extended period of time it can help to build the trust and rapport which is essential in this type of study. Additionally, depending on the views of participants, small body worn cameras may be offered to staff.

#### Process

**Fig 2:**
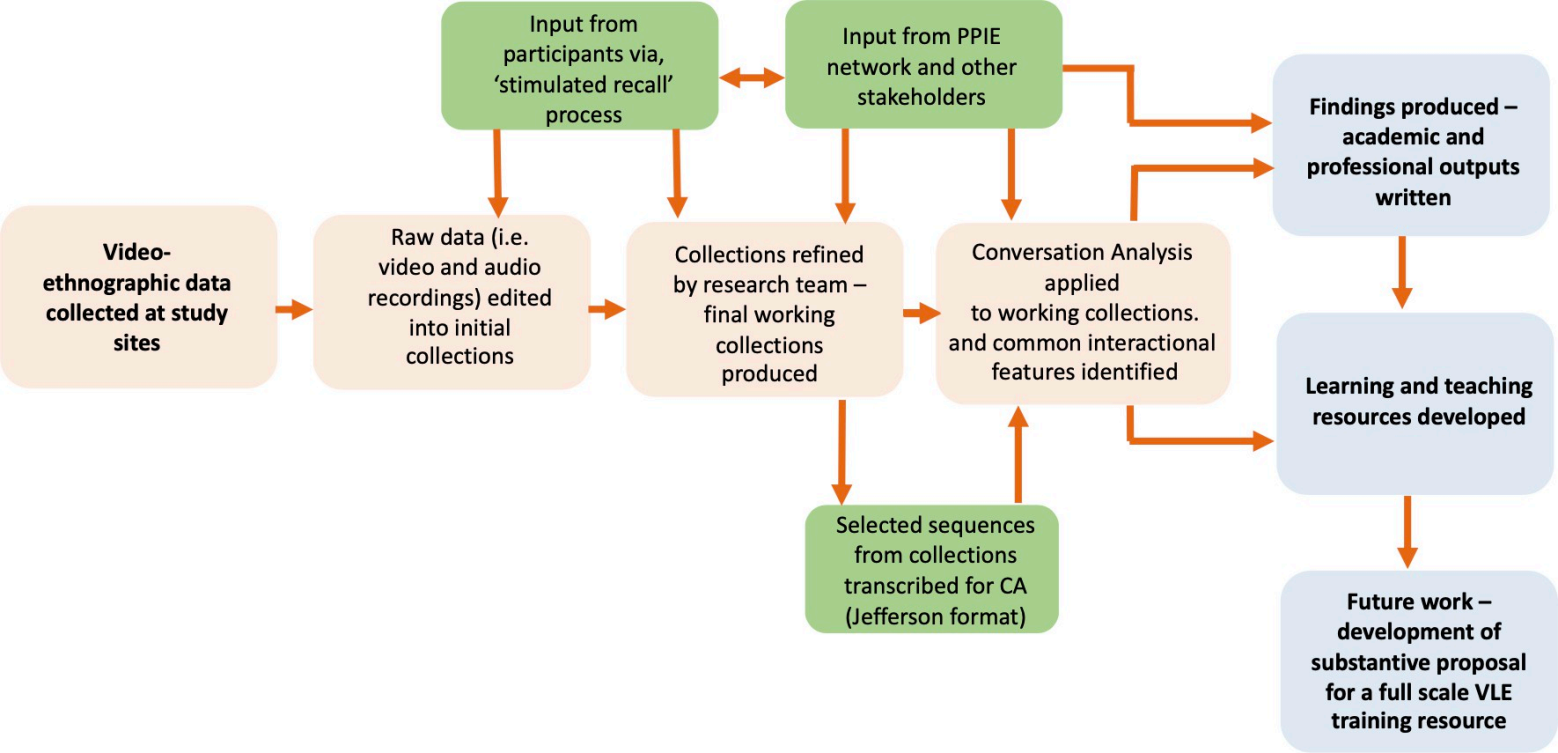
Data collection, analytical process and outputs.

Fieldwork, data collection, and elements of preliminary analysis will be closely connected, and at times concurrent (See Fig 2).

- During fieldwork, researchers will monitor the areas being covered by cameras and make field notes on instances of potentially ‘difficult’ encounters as they occur. These observations will be recorded in a format which can be cross-referenced to the time-code of the video.
- After each fieldwork session, video-data will be reviewed by the researchers. Adobe CC video production software will be used to edit the material into collections of relevant clips. Additional material such as observations made during filming, or data from any ad hoc stimulated recall interviews that occurred will be attached to the video files as meta data.
- At regular points during the fieldwork phase, edited collections of clips will be analysed thematically in more detail, with input from the wider research team and project Advisory Group, to develop and categorise the data into different types of difficult or challenging interactions.
- As collections of relevant material take shape, selected sequences will be transcribed using a standard (Jefferson) CA format in preparation for the final analysis phase.
- Participants will be invited to take part in informal interviews and ad hoc ‘stimulated recall’ (SR) sessions. Utilising ongoing SR-based feedback from participants a final working collection will be produced, based around an emerging core set of themes.
- It is anticipated that the main data set will contain up to 400 hours of ‘raw’ video.

### Phase 3: Final (CA) analysis

#### Months 10–15

The analysis phase will involve a staged approach: all of the video-ethnographic, SR, preliminary CA data, additional observational data and field notes will be cross-referenced and integrated using Nvivo software. In-depth CA-based analysis will finally be conducted on transcription data relating to the core themes.

### Phase 4: Writing up and dissemination

#### Months 16–18

The final phase of the study will be spent working with participants to produce preliminary (video-based) training resources based on the findings of the study. A research proposal for follow-on funding to develop these will also be written. Two main academic papers are planned; one outlining the innovative methodological aspects of the study, and a main findings paper. Accessible articles specifically aimed at dementia care workers, family carers and other relevant stakeholder groups will be produced. Building on our previous patient and participant involvement (PPI) work in this area, where possible, these will be written collaboratively with study participants.

### Project and data management

The project is being sponsored by Midlands Partnership NHS Foundation Trust (MPFT) and has undergone a Data Protection Impact Assessment (DPIA). Anonymised audio, video and text data will be managed in accordance with MPFT (NHS) Research and Governance requirements.
